# Novel Alleles of Two Tightly Linked Genes Encoding Polygalacturonase-Inhibiting Proteins (VrPGIP1 and VrPGIP2) Associated with the *Br* Locus That Confer Bruchid (*Callosobruchus* spp.) Resistance to Mungbean (*Vigna radiata*) Accession V2709

**DOI:** 10.3389/fpls.2017.01692

**Published:** 2017-09-28

**Authors:** Anochar Kaewwongwal, Jingbin Chen, Prakit Somta, Alisa Kongjaimun, Tarika Yimram, Xin Chen, Peerasak Srinives

**Affiliations:** ^1^Department of Agronomy, Faculty of Agriculture at Kamphaeng Saen, Kasetsart University, Bangkok, Thailand; ^2^Center for Advanced Studies for Agriculture and Food, Kasetsart University Institute for Advanced Studies, Kasetsart University, Bangkok, Thailand; ^3^Institute of Industrial Crops, Jiangsu Academy of Agricultural Sciences, Nanjing, China; ^4^Faculty of Animal Sciences and Agricultural Technology, Silpakorn University, Cha-Am, Thailand

**Keywords:** bruchid, seed weevil, polygalacturonase inhibitor, PGIP, *Callosobruchus*, insect resistance, mungbean

## Abstract

Nearly all mungbean cultivars are completely susceptible to seed bruchids (*Callosobruchus chinensis* and *Callosobruchus maculatus*). Breeding bruchid-resistant mungbean is a major goal in mungbean breeding programs. Recently, we demonstrated in mungbean (*Vigna radiata*) accession V2802 that *VrPGIP2*, which encodes a polygalacturonase inhibiting protein (PGIP), is the *Br* locus responsible for resistance to *C. chinensis* and *C. maculatus*. In this study, mapping in mungbean accession V2709 using a BC_11_F_2_ population of 355 individuals revealed that a single major quantitative trait locus, which controlled resistance to both *C. chinensis* and *C. maculatus*, was located in a 237.35 Kb region of mungbean chromosome 5 that contained eight annotated genes, including *VrPGIP1* (*LOC106760236*) and *VrPGIP2* (*LOC106760237*). *VrPGIP1* and *VrPGIP2* are located next to each other and are only 27.56 Kb apart. Sequencing *VrPGIP1* and *VrPGIP2* in “V2709” revealed new alleles for both VrPGIP1 and VrPGIP2, named *VrPGIP1-1* and *VrPGIP2-2*, respectively. *VrPGIP2-2* has one single nucleotide polymorphism (SNP) at position 554 of wild type *VrPGIP2*. This SNP is a guanine to cystine substitution and causes a proline to arginine change at residue 185 in the VrPGIP2 of “V2709”. *VrPGIP1-1* has 43 SNPs compared with wild type and “V2802”, and 20 cause amino acid changes in VrPGIP1. One change is threonine to proline at residue 185 in VrPGIP1, which is the same as in VrPGIP2. Sequence alignments of VrPGIP2 and VrPGIP1 from “V2709” with common bean (*Phaseolus vulgaris*) PGIP2 revealed that residue 185 in VrPGIP2 and VrPGIP1 contributes to the secondary structures of proteins that affect interactions between PGIP and polygalacturonase, and that some amino acid changes in VrPGIP1 also affect interactions between PGIP and polygalacturonase. Thus, tightly linked *VrPGIP1* and *VrPGIP2* are the likely genes at the *Br* locus that confer bruchid resistance in mungbean “V2709”.

## Introduction

Mungbean [*Vigna radiata* (L.) Wilczek] is an important legume crop of Asia. It is cultivated on ~6 million hectares, mainly in Asia (Somta et al., 2007). The crop is popularly grown for seeds after rice or wheat because of its early maturity (60–75 day), drought tolerance and symbiotic relationship with rhizobia, which fix atmospheric nitrogen to the soil. Seeds of mungbean contain high levels of proteins (20–25%) and carbohydrate (60–75%), and thus, they are an important human dietary staple (Somta et al., 2007).

Bruchids or seed weevils (Coleoptera: Bruchidae) are serious pests that damage legume seeds after harvest. Bruchids infest seeds in the field and after harvest, and the latter can results in total loss of a seed lot within 3–4 months (Srinives et al., 2007). The bruchid-damaged seeds are inedible and cannot be used for agricultural and commercial purposes. Azuki bean weevil (*Callosobruchus chinensis* L.) and cowpea weevil (*Callosobruchus muculatus* F.) are the major bruchid species that feed on seeds of mungbean and other food legumes, including cowpea (*Vigna unguiculata* L.), black gram [*Vigna mungo* (L.) Hepper], Bambara groundnut [*Vigna subterranea* (L.) Verdc.], azuki bean [*Vigna angularis* (Willd.) Ohwi and Ohashi], soybean [*Glycine max* (L.) Merr.], chickpea (*Cicer arietinum* L.) and pigeon pea [*Cajanus cajan* (L.) Millsp.]. Although *C. chinensis* and *C. maculatus* originated in Asia and Africa, respectively, they are currently present in almost every continent owing to international seed/grain trading. Generally, bruchids are controlled by chemical fumigation and dusting. However, these chemicals are not only harmful to humans and the environment, but also increase production costs. Additionally, chemical control is not practical for small-landholder farmers. Thus, a major goal in mungbean breeding programs is to develop bruchid-resistant cultivar(s) (Somta et al., 2007; Srinives et al., 2007).

Several bruchid-resistant mungbean germplasms have been identified. Fujii and Miyazaki (1987) reported that wild mungbean (*V. radiata* var. *sublobata*) accession TC1966 is completely resistant to *C. chinensis* and *C. maculatus*. Talekar and Lin (1992) found that cultivated mungbean accessions V2709 and V2802 are highly resistant to *C. chinensis*. Somta et al. (2008) demonstrated that V2709 and V2802 are also highly resistant to *C. maculatus*, while the cultivated mungbean accessions V1128 and V2817 are completely resistant to both bruchids.

Kitamura et al. (1988) showed that resistance to *C. chinensis* in “TC1966” is controlled by a single dominant gene, *Br*. Somta et al. (2007) demonstrated that resistance to *C. chinensis* and *C. maculatus* in “V2709” and “V2802” is controlled by a single dominant gene with some modifiers. Kaga and Ishimoto (1998) fine mapped the *Br* gene in “TC1966” to a region of 0.7 cM between restriction fragment length polymorphism (RFLP) markers Bng110 and Bng143. The *Br* gene is only 0.2 cM away from the Bng143. Chotechung et al. (2011) found that expressed sequence tag-simple sequence repeat (EST-SSR) marker DMB-SSR158 (DMB158 in the original report) co-segregated perfectly with the *Br* gene in “V2802”. Later, Chotechung et al. (2016) narrowed the genome region of the *Br* gene to a 38-kb segment on chromosome 5 and found that a gene, *VrPGIP2*, encoding polylacturonase-inhibiting protein (PGIP; also known as polylacturonase inhibitor) was probably responsible for the bruchid resistance. They also showed that “V2802”, “V1128”, “V2817”, and “TC1966” had the same *VrPGIP2* allele.

For “V2709”, the mode of bruchid-resistance inheritance is the same as for “V2808” and “TC1966” (Somta et al., 2007). However, the EST-SSR marker DMB-SSR158, which is located on the *VrPGIP2* sequence, did not reveal a polymorphism between “V2709” and bruchid-susceptible mungbean “Kamphaeng Saen 1” (KPS1; Chotechung et al., 2011), which was the same susceptible parent used by Chotechung et al. (2016). This suggested that the gene or allele for bruchid resistance in “V2709” is different from that of “V2802”.

In this study, we report the identification of new alleles for bruchid resistance in mungbean accession V2709. The objectives of this study were to (i) fine map the *Br* locus in “V2709”, (ii) identify candidate gene(s) for the *Br* locus in “V2709” and (iii) identify mutation(s) responsible for the resistance.

## Materials and methods

### Plant population and DNA extraction

A BC_11_F_2_ mapping population was developed from “KPS1” and “V2709” (Supplementary Figure S1). “KPS1” is susceptible to *C. chinensis* and *C. maculatus*, while “V2709” is resistant to both bruchids (Somta et al., 2008). In total, 355 BC_11_F_2_ plants together with “KPS1” and “V2709” were planted in an experimental field of Kasetsart University, Kamphaeng Saen Campus, Nakhon Pathom, Thailand from February to April 2013. Seeds from each BC_11_F_2_ plant were harvested for bruchid-resistance evaluation. Genomic DNA from all of the mungbean plants was extracted from young leaf tissues using a modified cetyl trimethylammonium bromide (CTAB) method (Lodhi et al., 1994).

### Evaluation for bruchid resistance

Cultures of *C. chinensis* and *C. maculatus* were reared on “KPS1” seeds. The resistance evaluation was carried out as per Somta et al. (2007) with minor modifications. In brief, 30 to 40 intact seeds from each BC_11_F_2_ plant were placed into a transparent plastic box. Twenty pairs (males and females) of 1- to 3-day-old bruchids were introduced into the box, kept for 7 day for egg laying, and then removed from the box. Parental seeds were tested and replicated five times. The seeds were maintained at 28°C and 70% relative humidity. Then, 60 day after insect introduction, the numbers of damaged seeds were counted and the percentages calculated.

### Segregation analysis

The monogenic inheritance of bruchid resistance in “V2709” (Somta et al., 2007; Chotechung et al., 2011) was confirmed. BC_11_F_2_ plants were classified into two classes; plants with 0–80%-damaged seeds were classified as resistant, which included the homozygous resistant genotype (highly resistant, with 0–20%-damaged seeds) and heterozygous resistant genotype (moderately resistant, with 21–80%-damaged seeds), while plants with 81–100%-damaged seeds were classified as the homozygous susceptible genotype (Somta et al., 2007). A chi-square (χ^2^) test was conducted to determine a 3 (resistance):1 (susceptible) goodness of fit using software *R*-program 2.0.10 (R Development Core Team, 2012).

### DNA marker analysis

In total, 25 DNA markers were developed based on the chromosome 5 sequence of mungbean (Supplementary Table S1). These markers, together with 52 SSR and sequence-tagged site (STS) markers located on mungbean chromosome 5 associated with the bruchid resistance reported by Chotechung et al. (2011, 2016), Hong et al. (2015), and Liu et al. (2016), were used to detect polymorphisms between “KPS1” and “V2709” (Supplementary Table S1). PCR amplification, gel electrophoresis and DNA band visualization were carried out as per Somta et al. (2009). SSR markers showing polymorphisms between “KPS1” and “V2709” were used to genotype the BC_11_F_2_ plants.

### Quantitative trait locus (QTL) analysis

A genetic linkage map was constructed using software QTL IciMapping 4.0 (Meng et al., 2015). Markers were grouped by a log of odds (LOD) value of 3.0. The recombination frequency between markers was converted into genetic map distances (centimorgan; cM) using the Kosambi mapping function (Kosambi, 1944).

The QTL for bruchid resistance was mapped using the inclusive composite interval mapping (ICIM) method (Li et al., 2007) by QTL IciMapping 4.0. The significant LOD threshold for the QTL was determined by a 5,000 permutation test at *P* = 0.01. ICIM was performed at 1-cM steps, and the *P*-value for entering variables (PIN) was 0.001.

### Sequencing of *VrPGIP1* and *VrPGIP2* in “V2709”

*VrPGIP2* in “V2709” was amplified and sequenced following the procedures described by Chotechung et al. (2016). *VrPGIP1* was amplified and sequenced in “KPS1” and “V2802” using the primer pair Br_03940_F1 (5′-CGACTAGCACCGGAAATTA-3′)/Br_03940_R13 (5′-CTCAACTTGGTTAATGATGCTTA-3′), and in “V2709” using the primer pair Br_03940_F1 (5′-CGACTAGCACCGGAAATTA-3′)/Br_03940_R14 (5′- ACACTGCACACGTGTCAAA−3′). PCR amplification and sequencing protocols were the same as those for *VrPGIP2*. NCBI nucleotide accession numbers for *VrPGIP1* and *VrPGIP2* are MF398961 and MF398954, respectively, for “KPS1”, MF496138 and MF398959, respectively, for “V2709”, and MF398960 and MF398955, respectively, for “V2802”.

The sequences of *VrPGIP1* and *VrPGIP2* from “KPS1”, “V2709”, and “V2802” were aligned using ClustalW (Larkin et al., 2007). The predicted protein sequences of both genes in these accessions were also aligned to detect protein sequence variation. In addition, the amino acids of *VrPGIP1* and *VrPGIP2* were aligned with PvPGIP2 (P58822.1), which is the only PGIP that has a known crystal structure (Di Matteo et al., 2003), to determine their primary protein structures.

### Phylogenetic analysis of PGIPs

VrPGIP1 and VrPGIP2 sequences and PGIP sequences of chickpea (*C. arietinum* L.) [CaPGIP1 (XP_004504732.1) and CaPGIP2 (XP_012572409.1)], common bean (*Phaseolus vulgaris* L.) [PvPGIP1 (CAI11357.1), PvPGIP2 (P58822.1), PvPGIP3 (CAI11359.1) and PvPGIP4 (CAI11360.1)], soybean (*G. max* Merr.) [GmPGIP1 (CAI99392.1), GmPGIP2 (CAI99393.1), GmPGIP3 (CAI99394.1), GmPGIP4 (CAI99395.1), GmPGIP5 (XP_003524769.1) and GmPGIP7 (XP_003531070.1)] and *Medicago truncatula* [MtPGIP1 (XP_013457276.1), MtPGIP2, (XP_013457278.1) and MtPGIP3 (XP_013457280.1)] were subjected to phylogenic analysis using software Phylogeny.fr (Dereeper et al., 2008; http://www.phylogeny.fr).

## Results

### Bruchid resistance in the BC_11_F_2_ population

**Figure 1:**
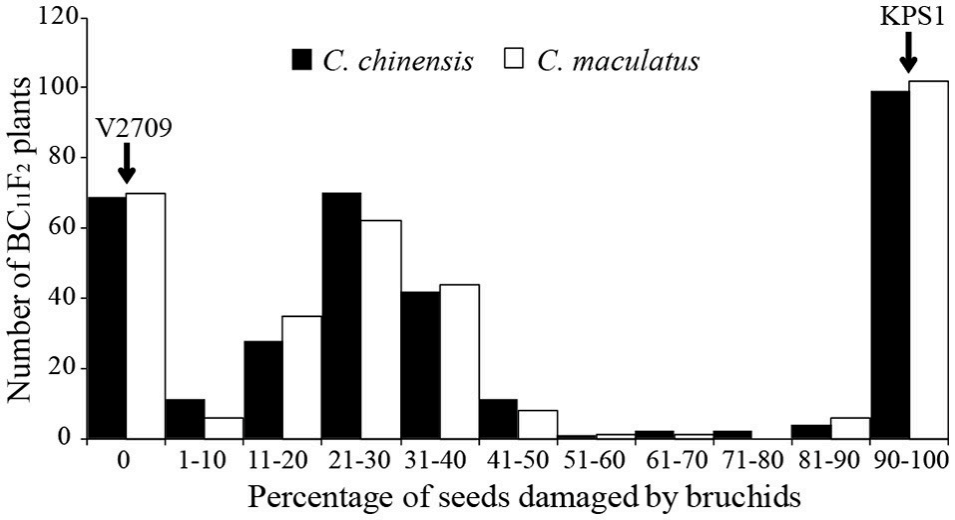
Frequency distribution of the percentage of seeds damaged by *Callosobruchus chinensis* and *Callosobruchus maculatus* in the mungbean BC_11_F_2_ population derived from “KPS1” and “V2709” as recipient and donor parents, respectively.

Seeds of “V2709” were free from damage by *C. chinensis* and *C. maculatus*, while seeds of “KPS1” were all damaged by both bruchid species. In the BC_11_F_2_ population, the level of seed's damaged ranged from 0 to 100%, with means of 44.19% for *C. chinensis* and 44.60% for *C. maculatus*. There was a very high correlation (*r* = 0.97, d.f. = 317, *P* < 0.0001) between the percentages of damaged seeds caused by the two bruchid species, suggesting that the resistance to *C. chinensis* and to *C. maculatus* in “V2709” is controlled by the same gene(s). The frequency distribution of the percentage of damaged seeds was nearly discontinuous and bimodal (Figure 1); however, there was a clear continuous distribution for the resistance group (1–50%-damaged seeds).

The segregation of plants in the BC_11_F_2_ population that showed resistance and susceptibility to *C. chinensis* and *C. maculatus* did not fit the 3 (resistant):1 (susceptible) ratio (χ^2^ = 5.24, *P* = 0.0221 and χ^2^ = 9.36, *P* = 0.0022, respectively). This result contrasted previous findings reported by Somta et al. (2007) and Chotechung et al. (2011) that a single dominant gene, *Br*, controls bruchid resistance in “V2709”. This contradiction may result from our BC_11_F_2_ population being a composite population derived from six BC_11_F_2_ families each having between 41 and 75 plants. Families with small number of plants may cause the distortion of *Br* locus-associated resistance in the composite population. In addition, modifying factors for the resistance presented in “V2709” (Somta et al., 2007) may also cause the distortion in resistance segregation.

### QTL mapping for bruchid resistance in “V2709”

In total, 77 DNA markers located near the *Br* locus on chromosome 5 of mungbean were screened for polymorphisms between “V2709” and “KPS1”, and 19 polymorphic markers were selected to construct a linkage map with a length of 84.12 cM.

A single major QTL for *C. chinensis* (*qBrc5.1*) and a single major QTL for *C. maculatus* (*qBrm5.1*) were detected by ICIM (Table 1 and Supplementary Figure S2). *qBrc5.1* and *qBrm5.1* were both located at 34.0 cM, between markers VrID1 and Vr-SSR017. These QTLs accounted for 95.58 and 96.30% of the total variation of seeds damage caused by *C. chinensis* and *C. maculatus*, respectively. The additive and dominant effects of *qBrc5.1* were 49.07 and 45.00%, respectively, while those of *qBrm5.1* were 48.72 and 44.17%, respectively. At both QTLs, allele(s) from “V2709” decreased the seed damage caused by bruchids. Because *qBrc5.1* and *qBrm5.1* were at the same position and conferred similar genetic effects for the resistance, we considered them as the same locus and named it “*qBr5.1*”.

**Table 1:** QTL conferring resistance to bruchids (*Callosobruchus chinensis* and *Callosobruchus maculatus*) identified by inclusive composite interval mapping in the BC_11_F_2_ mungbean population [KPS1 × (KPS1 × V2709)].

**Bruchid speceis**	**QTL name**	**Position (cM)**	**LOD score**	**Interval markers**	**1-LOD support interval (cM)**	**PVE^a^ (%)**	**Additive effect**	**Dominance effect**
*C. chinensis*	*qRcc5.1*	34.0	252.17	VrID1–VrBr-SSR017	33.5–34.5	93.34	49.07	45.00
*C. maculatus*	*qRcm5.1*	34.0	263.16	VrID1–VrBr-SSR017	33.5–34.5	93.84	48.72	44.17

*The resistance was determined based on the percentage of seeds damaged by bruchids*.

a *Phenotypic variance explained by the QTL*.

**Figure 2:**
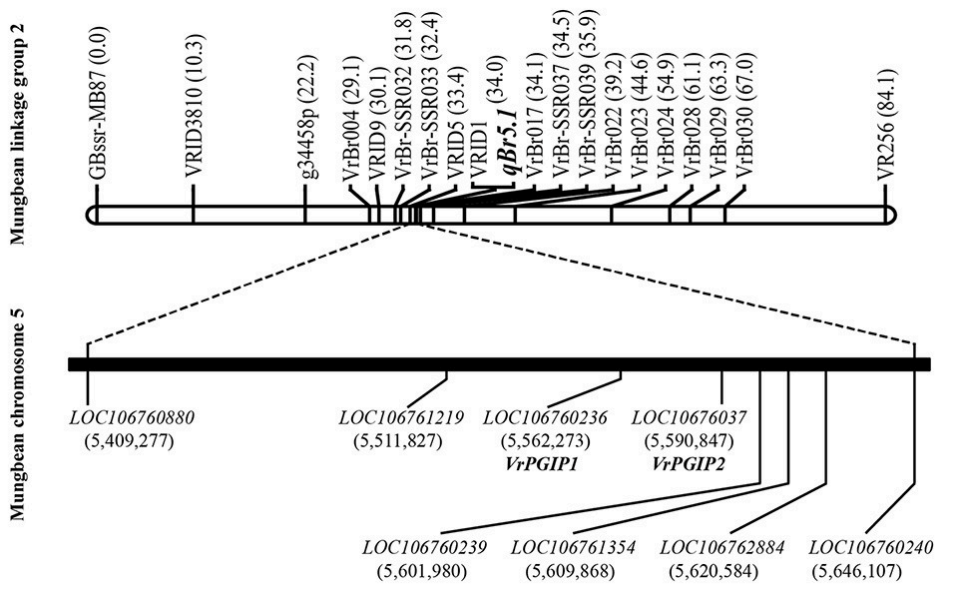
Location of the QTL *qBr5.1* controlling resistance to *Callosobruchus chinensis* and *Callosobruchus maculatus* in mungbean accession V2709. The QTL analysis was performed using BC_11_F_2_ population [KPS1 × (KPS1 × V2709)]. The 1-LOD confidence region that corresponds to chromosome 5 of the mungbean reference genome is shown. NCBI's annotated genes in the confidence region of the *qBr5.1* are also shown. Numbers in parenthesis after each marker indicate positions in centiMorgan (cM) units. Numbers in parenthesis under each gene indicate positions on mungbean chromosome 5.

The genomic confidence region of the *qBr5.1* was between markers VRID5 and VrBr-SSR037, covering ~237.35 Kb (Vr05: 5,410,272–5,647,621), in which there were eight annotated protein-encoding genes, including *VrPGIP1* (*LOC106760236*) and *VrPGIP2* (*LOC106760237*), based on the annotation by NCBI (Figure 2 and Supplementary Table S2).

### New alleles of *VrPGIP1* and *VrPGIP2* in “V2709”

**Figure 3:**
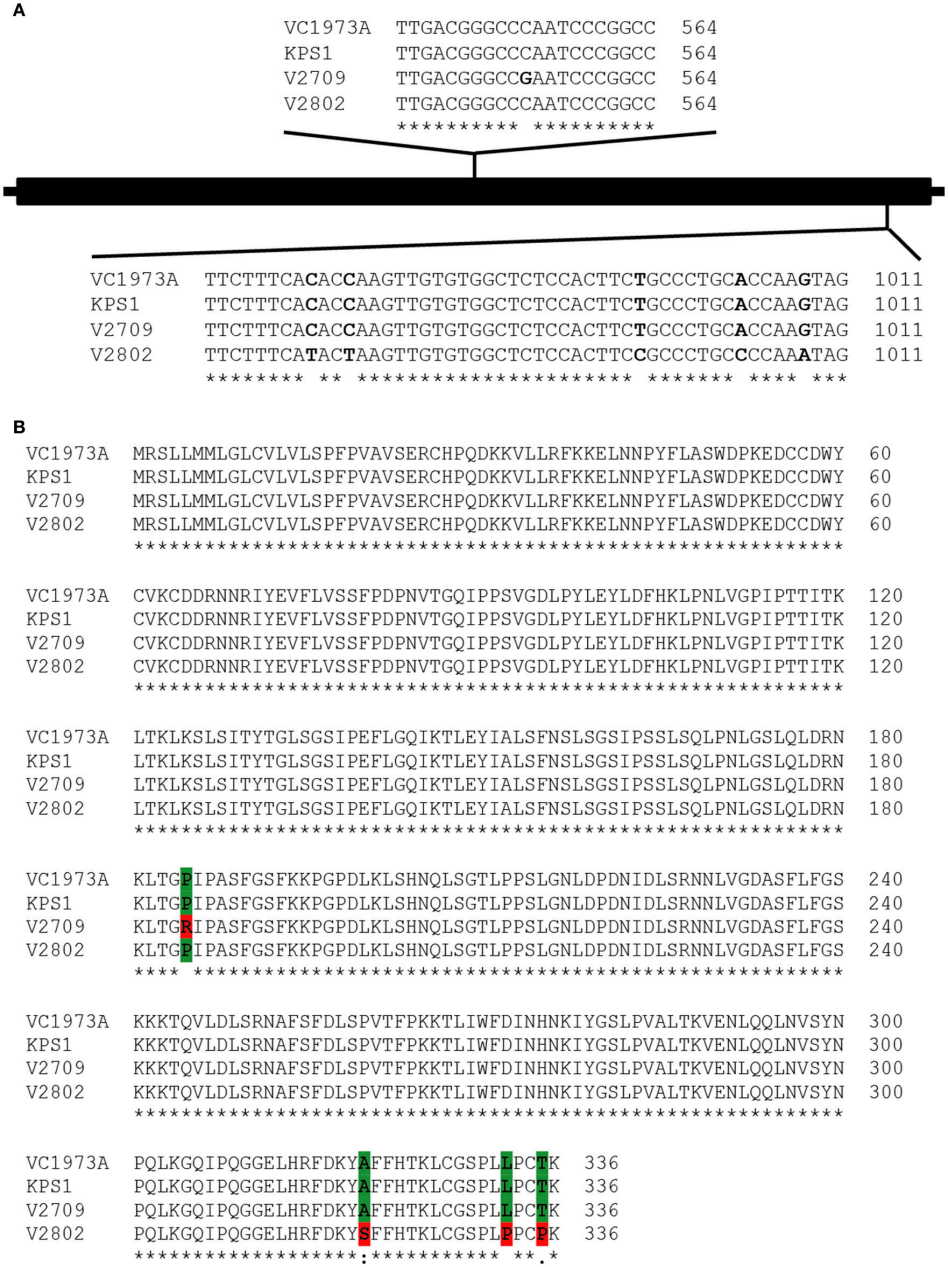
Single nucleotide polymorphisms in the *VrPGIP2* (LOC106760237) sequences from mungbean accession VC1973A (reference sequence), KPS1, V2709, and V2802. **(A)** “VC1973A” and “KPS1” are susceptible to bruchids, while “V2802” and “V2709” are resistant to bruchids. Polymorphic sites are presented in bold color. **(B)**The alignment of the protein sequences encoded by *VrPGIP2* in these mungbean accessions is also shown. Polymorphic sites are colored.

*VrPGIP2* is an intronless gene with 1,011 nucleotides, and it translates into a 336-amino acid protein (Chotechung et al., 2016). It is the same gene as *LOC106760237* annotated by NCBI. A sequence alignment of *VrPGIP2* genes from “V2802” and KPS1 showed seven SNPs (at nucleotide positions 573, 958, 969, 972, 995, 1,003 and 1,008) between the two mungbean accessions. The SNPs at positions 958, 995 and 1,003 caused amino acid changes in VrPGIP2 at positions 320, 332, and 335 in “V2802” (Chotechung et al., 2016). In this study, the sequencing of *VrPGIP2* in “V2709” revealed that *VrPGIP2* was 1,011 nucleotides, which was the same as in “V2802” and “KPS1”. A sequence alignment of *VrPGIP2* from “V2709” and “KPS1” revealed a single SNP at the 554 position (Figure 3A). The nucleotide at this position was cytosine in “KPS1” and guanine in “V2709”. This SNP caused an amino acid change at position 185(from arginine to proline) of the VrPGIP2 protein in “V2709” (Figure 3B). There were no polymorphisms between “V2802” and “KPS1” at this position (Figure 3A). We designated the *VrPGIP2* allele in “V2802” as *VrPGIP2-1* and that in “V2709” as *VrPGIP2-2*. We also aligned VrPGIP2 of “V2709” with PvPGIP2. The alignment showed that both VrPGIP2 and PvPGIP2 have 10 leucine-rich regions, and the amino acid change in VrPGIP2 of “V2709” is in leucine-rich region 5. The residue 185 of VrPGIP2, which is polymorphic between “V2709” and “KPS1” is equivalent to residue 193 of PvPGIP2 (**Figure 6A**).

**Figure 4:**
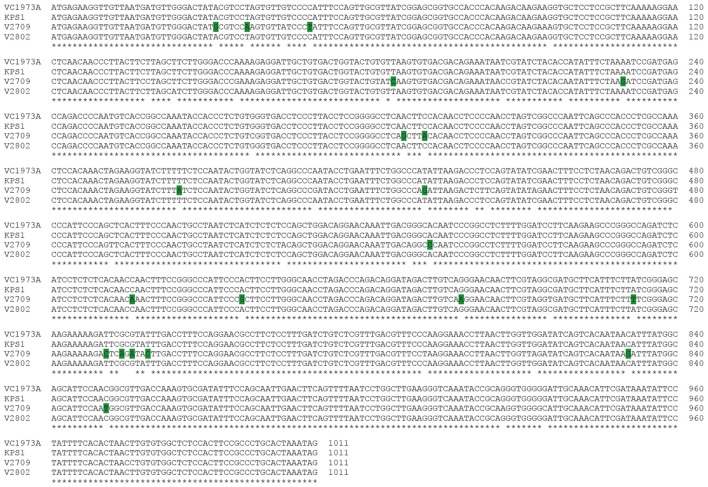
Alignment of the coding sequences of *VrPGIP1* (LOC106760236) from the accession VC1973A (reference sequence), KPS1, V2709, and V2802. “VC1973A” and “KPS1” are susceptible to bruchids, while “V2802” and “V2709” are resistant to bruchids. Polymorphic sites are colored.

**Figure 5:**
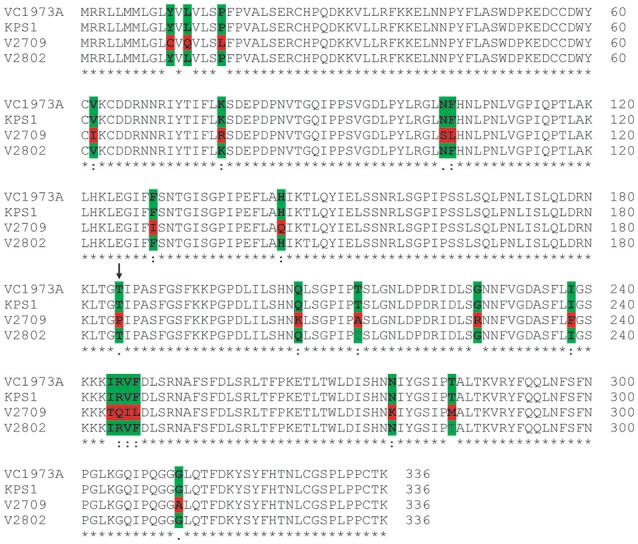
Alignment of the protein sequences encoded by *VrPGIP1* (LOC106760236) from the accession VC1973A (reference sequence), KPS1, V2709, and V2802. “VC1973A” and “KPS1” are susceptible to bruchids, while “V2802” and “V2709” are resistant to bruchids. Polymorphic sites are present in bold color. Arrow indicates position of important amino acid for formation of secondary structure of the VrPGIP protein and PG recognition.

*VrPGIP1* was annotated by Kang et al. (2014), and it had three exons with an open reading frame of 1,302 nucleotides that encodes a 433-amino acid protein (Chotechung et al., 2016). However, the current mungbean genome annotation by NCBI showed that *VrPGIP1* (*LOC106760236*) is intronless, has 1,011 nucleotides and encodes a 336-amino acid protein, which is the same as *VrPGIP2*. We conducted Sanger sequencing of this gene in “KPS1”, “V2709”, and “V2802”, and successfully obtained the ORF sequences for “KPS1” and “V2802” but we only obtained a continuous 960-bp sequence (including start codon) for “V2709”. We obtained the full sequence of *VrPGIP1* from our in-house whole genome sequence (WGS) data of “V2709”. However, 100% of the partial *VrPGIP1* sequence of “V2709” obtained from the Sanger sequencing was identical to the full *VrPGIP1* sequence generated from the WGS (data not shown). This indicated the high accuracy of the *VrPGIP1* sequence in “V2709” generated by the WGS. A sequence alignment of *VrPGIP2* genes from “V2709”, “V2802”, “KPS1” and the reference sequence (“VC1973A”) showed that the sequences from “KPS1”, “VC1973A”, and “V2802” were identical and that there were 43 SNPs between “V2709” and “KPS1” (Figure 4). Among those SNPs, 20 caused amino acid changes in “V2709”, when compared with “KPS1” (Figure 5). Interestingly, of those amino acid changes, the change at position 185 (from threonine to proline) was similar to the change in VrPGIP2. We named the *VrPGIP1* allele in “V2709” as *VrPGIP1*-*1*.

### Phylogeny of VrPGIP1 and VrPGIP2

A phylogenetic analysis was conducted to determine relationships of VrPGIP1 and VrPGIP2 with PGIPs from common bean, soybean, chickpea and *M. truncatula*. The phylogenetic tree revealed that VrPGIP1 and VrPGIP2 were closely related to each other but distantly related to the PGIPs from the other legumes (Supplementary Figure S3). Among the other legumes, VrPGIP1 and VrPGIP2 showed the closest relationship with MtPGIP3 of *M. truncatula*.

## Discussion

Although the wild mungbean accession TC1966, which carries the *Br* locus that confers resistance to *C. chinensis* and *C. maculatus*, has been reported since 1987 (Fujii and Miyazaki, 1987), only a few cultivars possessing the *Br* locus have been released to farmers (Lee et al., 2000; Yao et al., 2015). This is mainly due to skepticism about the safety of the *Br* locus. The chemical responsible for bruchid resistance at the *Br* locus is not known, and toxicity studies of the bruchid-resistant mungbeans carrying this locus are very limited (Miura et al., 1996; Yao et al., 2015). Kitamura et al. (1990) reported that the chemical involved in bruchid resistance was heat-stable and possibly a polysaccharide. Cyclo-peptide alkaloids, called Vignatic acid A and Vignatic acid B, isolated from a bruchid-resistant breeding line were detrimental to *C. chenensis* (Sugawara et al., 1996). A cysteine-rich protein designated VrCRP/VrD1 (defensin) identified from a bruchid-resistant breeding line inhibited the development of *C. chenensis* larva (Chen et al., 2002). However, gene mapping (Kaga and Ishimoto, 1998) and linkage analyses (Isemura et al., 2012) revealed that the loci controlling these chemicals are not in the *Br* locus.

Chotechung et al. (2016) demonstrated that *VrPGIP2*, which encodes a PGIP, is the candidate gene for the *Br* locus in mungbean “V2802”, although a role for *VrPGIP1*, which also encodes a PGIP and is located next to *VrPGIP2*, could not be eliminated. *VrPGIP2* in mungbean may confer bruchid resistance by inhibiting the digestive polygalacturonases (PGs) of bruchids that degrade pectin in the seeds, producing an energy source for the bruchids and helping other digestive enzymes to gain access to their substrates in bruchid food (Chotechung et al., 2016). In this study, we located a single major QTL for bruchid resistance with a high genetic effect (phenotypic variance explained (PVE) > 95%) on chromosome 5 using mungbean accession “V2709”. The genomic confidence region of the QTL (VrID5 to VrBr-SSR037) covered ~237.35 Kb (Vr05: 5,410,272–5,647,621) with eight protein-encoding genes, including *VrPGIP1* and *VrPGIP2* (Figure 1 and Supplementary Table S2).

**Figure 6:**
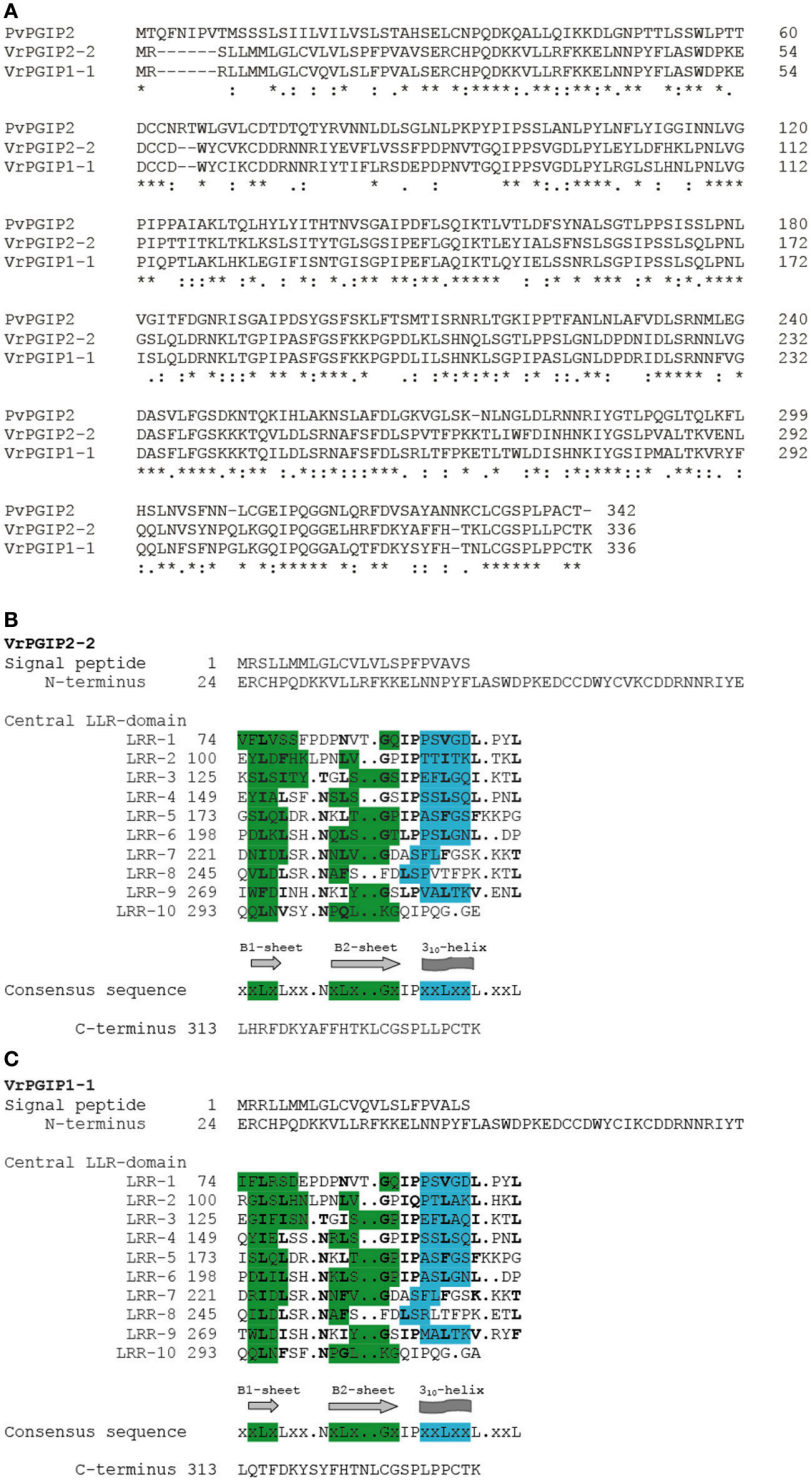
Alignment and putative secondary structures of VrPGIP2-2, VrPGIP1-1 and PvPGIP2 sequences. **(A)** Alignment of VrPGIP2-2, VrPGIP1-1 and PvPGIP2 sequences. PvPGIP2 is the only PGIP that secondary structure has been determined (Di Matteo et al., 2003). **(B)** Putative residues contributing to the secondary structure of VrPGIP2-2 and **(C)** of VrPGIP2-1 are based on PvPGIP2 and highlighted in green (Sheets B1 and Sheets B2) and blue (3_10_-helix).

We sequenced *VrPGIP1* and *VrPGIP2* in mungbean “V2709” and found new alleles, *VrPGIP1-1* and *VrPGIP2-2*, respectively (Figures 3A, **4**). The VrPGIP2 encoded by *VrPGIP2-2* possessed an amino acid change at the position 185, as compared with the wild type protein (Figure 3B). Comparing the sequence of VrPGIP2 with PvPGIP2, which is the only PGIP that has a known crystal structure, we found that residue 185 of the VrPGIP2 primary sequence (Figure 6B) corresponds to residue 164 of the crystal structure of PvPGIP2 (Di Matteo et al., 2003). Residue 164 is a site that contributes to the formation of the secondary structure of the PvPGIP2 protein (Di Matteo et al., 2003), and it is also a putative positive selection site of PvPGIP2 that plays an important role in PG recognition (Casasoli et al., 2009). The VrPGIP1 encoded by *VrPGIP1-1* showed many amino acid changes when compared with the wild type, and one change that occurred at position 185, which is the same position of the single amino acid change in VrPGIP2, resulted in the same amino acid at this position in VrPGIP1 and VrPGIP2 in “V2709” (Figures 5, 6C). PGs are important digestive enzymes found in the midgut of *Callosobruchus maculatus* (Pedra et al., 2003; Pauchet et al., 2010; Nogueira et al., 2012; Santana, 2013) and *Phaedon cochleariae* (mustard leaf beetle) (Kirsch et al., 2012), a coleopteran insect similar to the *Callosobruchus* species. A semi-purified PGIP from orange flavedo inhibited an endoPG purified from the larvae of sugarcane rootstalk borer weevil (*Diaprepes abbreviates*) (Doostdar et al., 1997), while PvPGIP3 and PvPGIP4 from the common bean strongly inhibited the PG activities of the mirid bugs *Lygus rugulipennis* and *Adelphocoris lineolatus* (D'Ovidio et al., 2004; Frati et al., 2006). Insect pectinase complexes, including PGs, are mainly found in Coleopteran and Hemipteran insects and are involved in plant penetration, the softening of plant material, and the digestion of young plant tissues and grains (Calderón-Cortés et al., 2012). Kirsch et al. (2016) showed that the rice weevil *Sitophilus oryzae*, a coleopteran insect that infests both cereal and legume seeds, uses PGs and pectin methylesterases synergistically to degrade pectin. If this was also the case in *C. chinensis* and *C. maculatus, VrPGIP2-2* and also *VrPGIP2-1* could confer resistance to these bruchids by inhibiting the ability of PG to degrade pectin, which would result in the retardation and arrest of insect growth and development.

Hong et al. (2015) demonstrated that two closely linked QTLs (~7-cM apart) with similar effects (PVE for each QTL was ~45%) controlled *C. chinesis* resistance in “V2709” although a genetic study revealed the monogenic inheritance of the trait. This contradicts our finding that only one single major QTL with a PVE greater than 98% on mungbean chromosome 5 controlled bruchid resistance in “V2709”. It is difficult to compare the results between the two studies because only one marker (GBssr-MB87) is common and it is far from the QTLs in both studies. Although the RP marker that is closely linked to a QTL reported by Hong et al. (2015) showed a polymorphism between the parents of our mapping population, it produced weak DNA bands that were difficult to unambiguously score. Nonetheless, the probable location of RP on chromosome 5 is between 5,210,000 and 5,520,000 (Chotechung et al., 2016).

In addition to *VrPGIP* genes that show an association with bruchid resistance (this study and Chotechung et al., 2011), *g39185* encoding a BURP domain-containing protein has been identified as a candidate gene for bruchid resistance in mungbean. *g39185* was identified by comparing transcript and protein profiles between bruchid-susceptible and bruchid-resistant mungbean, which derived its resistance from “TC1966” (Lin et al., 2016). *g39185* is at position 5,236,101 of mungbean chromosome 5 (Lin et al., 2016) which is ~326.7 and ~354.7 Kbp from *VrPGIP1* and *VrPGIP2*, respectively, and is very near the possible location of the RP marker reported by Hong et al. (2015). BURP domain-containing proteins have only been found in plants. Although many BURP proteins have been isolated from plants, their expression patterns are diverse and some functions are still unknown. Generally, BURP domain-containing proteins play important roles in maintaining normal plant metabolism or development (Shao et al., 2011; Tang et al., 2014; Li et al., 2016). Based on the information reported by Lin et al. (2016), *g39185* corresponds to *Vradi05g03810* on the reference mungbean chromosome 5, and *Vradi05g03810* is annotated as *LOC106759697* in the GenBank database. *LOC106759697* encodes a PG (https://www.ncbi.nlm.nih.gov/gene/?term$=$LOC106759697). Three forms of PGs were predicted from *LOC106759697*. A BLASTN algorithm-based search revealed that the gene was also most similar to a gene from azuki bean (e-value = 0.0, identity = 95%) that encodes a RESPONSIVE TO DEHYDRATION22 (RD22)-like protein of the BURP family. The RD22 protein is responsive to abiotic stresses, such as drought and salinity (Abe et al., 2003; Hanana et al., 2008). Based on the known and/or possible physiological function of PGIP and our results from gene sequencing and mapping in this study and in Chotechung et al. (2016), *VrPGIP2* and/or *VrPGIP1* is most likely the *Br* locus. Nonetheless, our results in this study indicated that the tightly linked genes *VrPGIP1* and *VrPGIP2* are likely the genes at the *Br* locus that confer bruchid resistance in the mungbean “V2709”. In addition, the different genetic bases of the bruchid resistance under the *Br* loci in “V2709” and “V2802” provides plant breeders the opportunity to develop new mungbean cultivar(s) with durable resistance to bruchids by pyramiding or rotating the *VrPGIP1* and *VrPGIP2* resistance genes.

## Author contributions

AnK and AlK carried out DNA marker analysis and data analysis. PrS, AnK, TY, and PeS prepared plant materials and conducted insect resistance evaluation. JC, XY, and XC performed DNA sequencing, and analyzed sequencing results. PrS initiated and coordinated the study, and analyzed the results. AnK, JC, PrS, XC, and PeS wrote the manuscript. All authors approved the manuscript.

### Conflict of interest statement

The authors declare that the research was conducted in the absence of any commercial or financial relationships that could be construed as a potential conflict of interest.

## References

[B1] Abe H. Urao T. Ito T. Seki M. Shinozaki K. Yamaguchi-Shinozaki K. (2003). Arabidopsis AtMYC2 (bHLH) and AtMYB2 (MYB) function as transcriptional activators in abscisic acid signaling. Plant Cell 15, 63–78. 10.1105/tpc.006130 12509522 PMC143451

[B2] Calderón-Cortés N. Quesada M. Watanabe H. Cano-Camacho H. Oyama K. (2012). Endogenous plant cell wall digestion: a key mechanism in insect evolution. Annu. Rev. Ecol. Evol. Syst. 43, 45–71. 10.1146/annurev-ecolsys-110411-160312

[B3] Casasoli M. Federici L. Spinelli F. Di Matteo A. Vella N. Scaloni F.. (2009). Integration of evolutionary and desolvation energy analysis identifies functional sites in a plant immunity protein. Proc. Natl. Acad. Sci. U.S.A. 106, 7666–7671. 10.1073/pnas.0812625106 19372373 PMC2678593

[B4] Chen K. C. Lin C. Y. Kuan C. C. Sung H. Y. Chen C. S. (2002). A novel defensin encoded by a mungbean cDNA exhibits insecticidal activity against bruchid. J. Agric. Food Chem. 50, 7258–7263. 10.1021/jf020527q 12452641

[B5] Chotechung S. Chankaew S. Srinives P. Somta P. (2011). Identification of DNA markers associated with bruchid resistance in mungbean. Khon Khan Agric. J. 39, 221–226.

[B6] Chotechung S. Somta P. Chen J. Yimram T. Chen X. Srinives P. (2016). A gene encoding a polygalacturonase-inhibiting protein (PGIP) is a candidate gene for bruchid (Coleoptera: bruchidae) resistance in mungbean (*Vigna radiata*). Theor. Appl. Genet. 129, 1673–1683. 10.1007/s00122-016-2731-1 27220975

[B7] Dereeper A. Guignon V. Blanc G. Audic S. Buffet S. Chevenet F.. (2008). Phylogeny.fr: robust phylogenetic analysis for the non-specialist. Nucleic Acids Res. 36, W465–W469. 10.1093/nar/gkn180 18424797 PMC2447785

[B8] Di Matteo A. Federici L. Mattei B. Salvi G. Johnson K. A. Savino C.. (2003). The crystal structure of polygalacturonase-inhibiting protein (PGIP)., a leucine-rich repeat protein involved in plant defense. Proc. Natl. Acad. Sci. U.S.A. 100, 10124–10128. 10.1073/pnas.1733690100 12904578 PMC187787

[B9] Doostdar H. McCollum T. G. Mayer R. T. (1997). Purification and characterization of an endo-polygalacturonase from the gut of West Indies sugarcane rootstalk borer weevil (*Diaprepes abbreviatus* L.) larvae. Comp. Biochem. Phys. B 118, 861–867. 10.1016/S0305-0491(97)00285-X

[B10] D'Ovidio R. Raiola A. Capodicasa C. Devoto A. Pontiggia D. Roberti S.. (2004). Characterization of the complex locus of bean encoding polygalacturonase-inhibiting proteins reveals subfunctionalization for defense against fungi and insects. Plant Physiol. 135, 2424–2435. 10.1104/pp.104.044644 15299124 PMC520809

[B11] Frati F. Galletti R. De Lorenzo G. Salerno G. Conti E. (2006). Activity of endo-polygalacturonases in mirid bugs (Heteroptera: Miridae) and their inhibition by plant cell wall proteins (PGIPs). Eur. J. Entomol. 103, 515–522. 10.14411/eje.2006.067

[B12] Fujii K. Miyazaki S. (1987). Infestation resistance of wild legumes (*Vigna sublobata*) to azuki bean weevil., *Callosobruchus chinensis* (L.) (Coleoptera: Bruchidae) and its relationship with cytogenetic classification. Appl. Entmol. Zool. 22, 229–230. 10.1303/aez.22.229

[B13] Hanana M. Deluc L. Fouquet R. Daldoul S. Léon C. Barrieu F.. (2008). Identification and characterization of “rd22” dehydration responsive gene in grapevine (*Vitis vinifera* L.). C. R. Biol. 331, 569–578. 10.1016/j.crvi.2008.05.002 18606386

[B14] Hong M. G. Kim K. H. Ku J. H. Jeong J. K. Seo M. J. Park C. H. (2015). Inheritance and quantitative trait loci analysis of resistance genes to bruchid and bean bug in mungbean (*Vigna radiata* L. Wilczek). Plant Breed. Biotechnol. 3, 39–46. 10.9787/PBB.2015.3.1.039

[B15] Isemura T. Kaga A. Tabata S. Somta P. Srinives P. Shimizu T.. (2012). Construction of a genetic linkage map and genetic analysis of domestication related traits in mungbean (Vigna radiata). PLoS ONE 7:e41304. 10.1371/journal.pone.0041304 22876284 PMC3410902

[B16] Kaga A. Ishimoto M. (1998). Genetic localization of a bruchid resistance gene and its relationship to insecticidal cyclopeptide alkaloids., the vignatic acids in mungbean (*V. radiata* L.Wilczek). Mol. Gen. Genet. 258, 378–384. 10.1007/s004380050744 9648742

[B17] Kang Y. J. Kim S. Kim M. Y. Lestari P. Kim K. H. Ha B. K.. (2014). Genome sequence of mungbean and insights into evolution within *Vigna* species. Nat. Commun. 5:5443. 10.1038/ncomms6443 25384727 PMC4241982

[B18] Kirsch R. Heckel D. G. Pauchet Y. (2016). How the rice weevil breaks down the pectin network: enzymatic synergism and sub-functionalization. Insect Biochem. Mol. 71, 72–82. 10.1016/j.ibmb.2016.02.007 26899322

[B19] Kirsch R. Wielsch N. Vogel H. Svatoš A. Heckel D. G. Pauchet Y. (2012). Combining proteomics and transcriptome sequencing to identify active plant-cell-wall-degrading enzymes in a leaf beetle. BMC Genomics 13:587. 10.1186/1471-2164-13-587 23116131 PMC3505185

[B20] Kitamura K. Ishimoto M. Ishii S. (1990). Bruchid resistance factors in Phaseolus and Vigna legumes, in Bruchids and Legumes: Economics., Ecology and Coevolution, eds. Fujii K. Gatehouse A. M. R. Johnson C. D. Mitchel R. Yoshida T. (Dordrecht: Kluwer Academic Publishers), 229–240.

[B21] Kitamura K. Ishimoto M. Sawa M. (1988). Inheritance of resistance to infestation with azuki bean weevil in *Vigna sublobata* and successful incorporation to *V. radiata*. Jap. J. Breed. 38, 459–464. 10.1270/jsbbs1951.38.459

[B22] Kosambi D. (1944). The estimation of map distances from recombination values. Ann. Eugen. 12, 172–175. 10.1111/j.1469-1809.1943.tb02321.x

[B23] Larkin M. A. Blackshields G. Brown N. P. Chenna R. McGettigan P. A. McWilliam H.. (2007). Clustal W and Clustal X version 2.0. Bioinformatics 23, 2947–2948. 10.1093/bioinformatics/btm404 17846036

[B24] Lee Y. H. Moon J. K. Park K. Y. Ku J. H. Yun H. T. Chung W. K. (2000). A new mungbean cultivar with bruchid resistance., “Jangannogdu”. Korean J. Breed. 32, 296–297. 10.1603/EC14113

[B25] Li H. Ye G. Wang J. (2007). A modified algorithm for the improvement of composite interval mapping. Genetics 175, 361–374. 10.1534/genetics.106.066811 17110476 PMC1775001

[B26] Li Y. Chen X. Chen Z. Cai R. Zhang H. Xiang Y. (2016). Identification and expression analysis of BURP domain-containing genes in *Medicago truncatula*. Front. Plant Sci. 7:485. 10.3389/fpls.2016.00485 27148311 PMC4829796

[B27] Lin W. J. Ko C. Y. Liu M. S. Kuo C. Y. Wu D. C. Chen C. Y.. (2016). Transcriptomic and proteomic research to explore bruchid-resistant genes in mungbean isogenic lines. J. Agric. Food Chem. 64, 6648–6658. 10.1021/acs.jafc.6b03015 27508985

[B28] Liu M. S. Kuo T. C. Y. Ko C. Y. Wu D. C. Li K. Y. Lin W. J. (2016). Genomic and transcriptomic comparison of nucleotide variations for insights into bruchid resistance of mungbean (*Vigna radiata* [L.] R. Wilczek). BMC Plant Biol. 16:46. 10.1186/s12870-016-0736-1 26887961 PMC4756517

[B29] Lodhi M. A. Ye G. N. Weeden N. F. Reisch B. I. (1994). A simple and efficient method for DNA extraction from grapevine cultivars and Vitis species. Plant Mol. Biol. Rep. 12, 6–13. 10.1007/BF02668658

[B30] Meng L. Li H. Zhang L. Wang J. (2015). QTL IciMapping: Integrated software for genetic linkage map construction and quantitative trait locus mapping in biparental populations. Crop J. 3, 269–283. 10.1016/j.cj.2015.01.001

[B31] Miura K. Ishimoto M. Yamanaka N. Miyazaki S. Hiramatsu M. Nakajima Y. (1996). Effects of bruchid-resistant mungbean meal on growth and blood-biochemical values in mice. JIRCAS J. 3, 23–31.

[B32] Nogueira F. C. S. Silva C. P. Alexandre D. Samuels R. I. Soares E. L. Aragão F. J. L.. (2012). Global proteome changes in larvae of *Callosobruchus maculatus* (Coleoptera: Chrysomelidae: Bruchinae) following ingestion of a cysteine proteinase inhibitor. Proteomics 12, 2704–2715. 10.1002/pmic.201200039 22833537

[B33] Pauchet Y. Wilkinson P. Chauhan R. Ffrench-Constant R. H. (2010). Diversity of beetle genes encoding novel plant cell wall degrading enzymes. PLoS ONE 5:e15635. 10.1371/journal.pone.0015635 21179425 PMC3003705

[B34] Pedra J. H. F. Brandt A. Westerman R. Lobo N. Li H. M. Romero-Severson J.. (2003). Transcriptome analysis of the cowpea weevil bruchid: identification of putative proteinases and alpha-amylases associated with food breakdown. Insect Mol. Biol. 12, 405–412. 10.1046/j.1365-2583.2003.00425.x 12864920

[B35] R Development Core Team (2012). R: A Language and Environment for Statistical Computing. Vienna: R Foundation for Statistical Computing Available online at: https://www.r-project.org/.

[B36] Santana M. C. (2013). Identificação de Poligalacturonases Expressas No Sistema Digestório de Callosobruchus Maculatus (Coleoptera: Chrysomelidae: Bruchinae). Thesis, Universidade Federal de Santa Catarina.

[B37] Shao Y. Wei G. Wang L. Dong Q. Zhao Y. Chen B.. (2011). Genome-wide analysis of BURP domain-containing genes in *Populus trichocarpa*. J. Integr. Plant Biol. 53, 743–755. 10.1111/j.1744-7909.2011.01068.x 21767343

[B38] Somta C. Somta P. Tomooka N. Ooi P. A. C. Vaughan D. A. Srinives P. (2008). Characterization of new sources of mungbean (*Vigna radiata* (L) Wilczek) resistance to bruchids., *Callosobruchus* spp (Coleoptera: Bruchidae). J. Stored Prod. Res. 44, 316–321. 10.1016/j.jspr.2008.04.002

[B39] Somta P. Ammaranan C. Ooi P. A. C. Srinives P. (2007). Inheritance of seed resistance to bruchids in cultivated mungbean (*Vigna radiata* (L) Wilzcek). Euphytica 155, 49–55. 10.1007/s10681-006-9299-9

[B40] Somta P. Seehalak W. Srinives P. (2009). Development, characterization and cross-species amplifcation of mungbean (Vigna radiata) genic microsatellite markers. Conserv. Genet. 10, 1939–1943. 10.1007/s10592-009-9860-x

[B41] Somta P. Srinives P. (2007). Genome research in mungbean [*Vigna radiata* (L.) Wilczek] and blackgram [*V. mungo* (L.) Hepper]. ScienceAsia 33(Suppl. 1), 69–74. 10.2306/scienceasia1513-1874.2007.33(s1).069

[B42] Srinives P. Somta P. Somta C. (2007). Genetics and breeding of resistance to bruchids (Callosobruchus spp) in Vigna crops: a review. *NU. Sci*. J. 4, 1–17.

[B43] Sugawara F. Ishimoto M. Le-Van N. Koshino H. Uzawa J. Yoshida S. (1996). Insecticidal peptide from mungbean: a resistant factor against infestation with azuki bean weevil. J. Agric. Food. Chem. 44, 3360–3364. 10.1021/jf960166c

[B44] Talekar N. S. Lin C. L. (1992). Characterization of *Callosobruchus chinensis* (Coleoptera: Bruchidae) resistance in mungbean. J. Econ. Entomol. 85, 1150–1153. 10.1093/jee/85.4.1150

[B45] Tang Y. Cao Y. Qiu J. Gao Z. Ou Z. Wang Y.. (2014). Expression of a vacuole-localized BURP-domain protein from soybean (SALI3-2) enhances tolerance to cadmium and copper stresses. PLoS ONE 9:e98830. 10.1371/journal.pone.0098830 24901737 PMC4047006

[B46] Yao Y. Cheng X. Ren G. (2015). A 90-day study of three bruchid-resistant mung bean cultivars in Sprague-Dawley rats. Food Chem. Toxicol. 76, 80–85. 10.1016/j.fct.2014.11.024 25533792

