# Supplementary material for: Novel Alleles of Two Tightly Linked Genes Encoding Polygalacturonase-Inhibiting Proteins (VrPGIP1 and VrPGIP2) Associated with the Br Locus That Confer Bruchid (Callosobruchus spp.) Resistance to Mungbean (Vigna radiata) Accession V2709
Source: Front Plant Sci. 2017 Sep 28;8:1692. doi: 10.3389/fpls.2017.01692 (PMC5625325; doi:10.3389/fpls.2017.01692)
Supplement: Supplementary file 2 [file Table2.PDF]

Supplementary Table S2. NCBI annotated genes residing between positions 5,410,272 to 5,647,621 of chromosome 5 of the reference mungbean sequence (VC1973A)

Gene	Gene description	Location on mungbean chromome 5
LOC106760880	dehydration-responsive protein RD22-like	5409277-5413301
LOC106761219	dehydration-responsive protein RD22-like	5511827-5514459
LOC106760236	polygalacturonase inhibitor-like	5562273-5563283
LOC106760237	polygalacturonase inhibitor-like	5590847-5591901
LOC106760239	AT-hook motif nuclear-localized protein 22-like	5601980-5602576
LOC106761354	uncharacterized protein	5609869-5610870
LOC106762884	probable alpha,alpha-trehalose-phosphate synthase [UDP-forming] 9	5620584-5625367
LOC106760240	spermidine synthase 1-like	5646107-5649419
