# Supplementary material for: Novel Alleles of Two Tightly Linked Genes Encoding Polygalacturonase-Inhibiting Proteins (VrPGIP1 and VrPGIP2) Associated with the Br Locus That Confer Bruchid (Callosobruchus spp.) Resistance to Mungbean (Vigna radiata) Accession V2709
Source: Front Plant Sci. 2017 Sep 28;8:1692. doi: 10.3389/fpls.2017.01692 (PMC5625325; doi:10.3389/fpls.2017.01692)

Supplementary Figure S1. A scheme showing development of $BC_{11}F_2$ population for bruchid resistance analysis from mungbean accessions Kamphaeng Saen 1 (KPS1; susceptible) and V2709 (resistant).

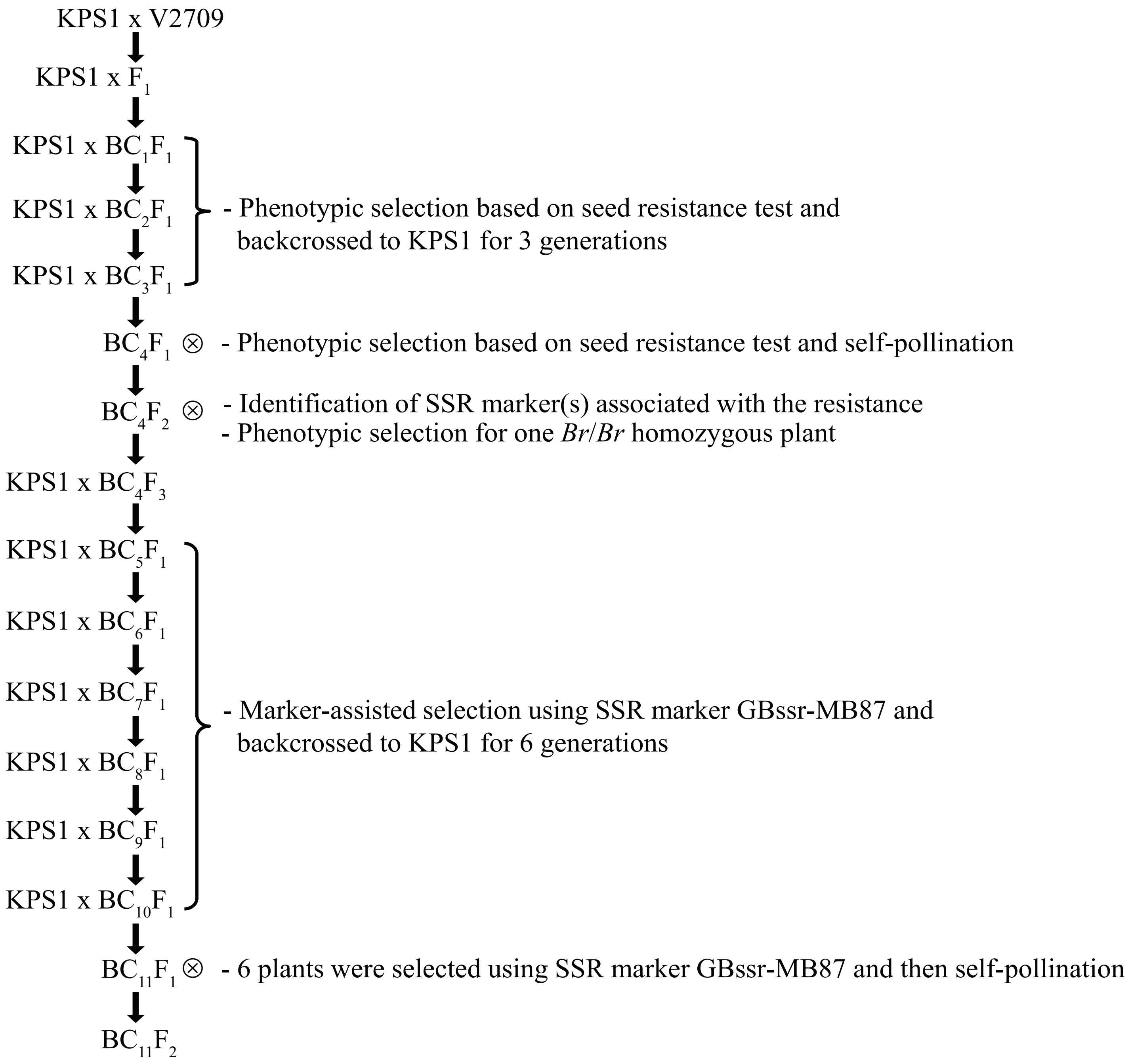

Supplement: Supplementary file 3 [file Image1.PDF]
