# Supplementary material for: Novel Alleles of Two Tightly Linked Genes Encoding Polygalacturonase-Inhibiting Proteins (VrPGIP1 and VrPGIP2) Associated with the Br Locus That Confer Bruchid (Callosobruchus spp.) Resistance to Mungbean (Vigna radiata) Accession V2709
Source: Front Plant Sci. 2017 Sep 28;8:1692. doi: 10.3389/fpls.2017.01692 (PMC5625325; doi:10.3389/fpls.2017.01692)
Supplement: Supplementary file 4 [file Image2.PDF]

Supplementary Figure S2. LOD-graph of the QTL for resistance to *Callosobruchus chinensis* (A) and *Callosobruchus maculatus* (B) detected in the $BC_{11}F_2$ population [KPS1 x (KPS1 x V2709)] by inclusive composite interval mapping method using QTL IciMapping software. LOD threshold calculated from 5,000 permutation test at P = 0.01 for the resistance to *Callosobruchus chinensis* is 3.63 and to *Callosobruchus maculatus* is 3.91.

A

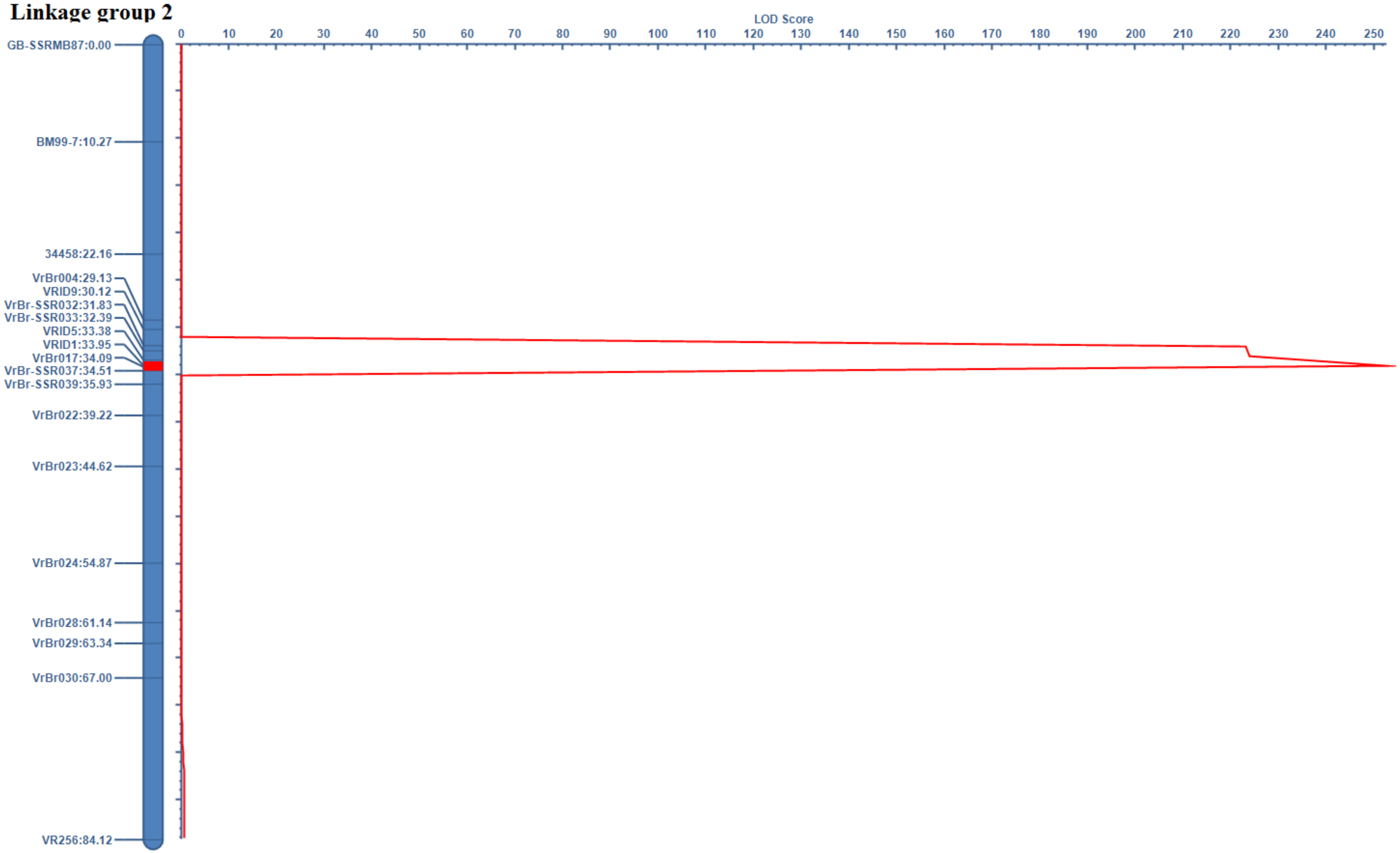

B

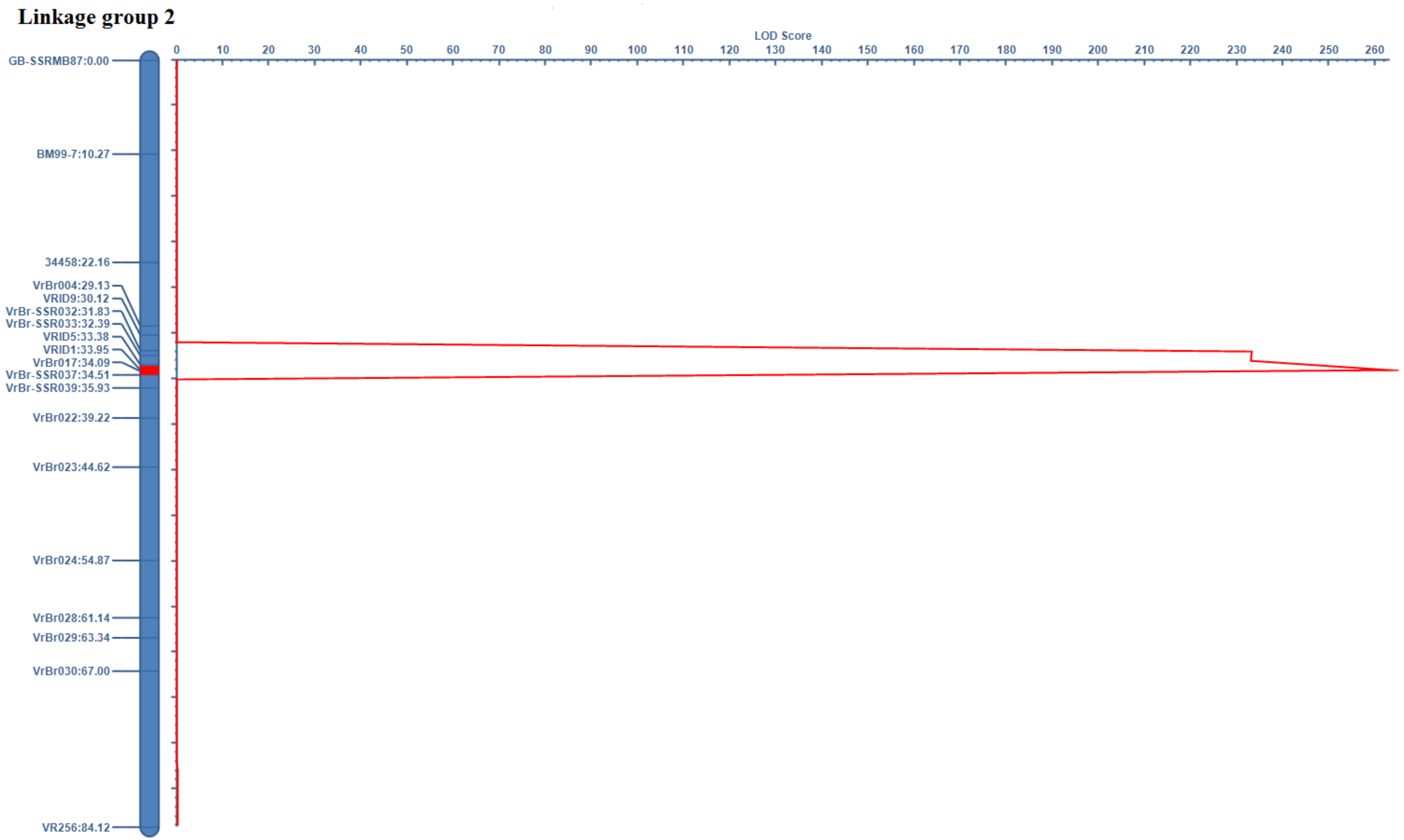
Linkage group 2
LOD Score
0
10
20
30
40
50
60
70
80
90
100
110
120
130
140
150
160
170
180
190
200
210
220
230
240
250
260
GB-SSRMB87:0.00
BM99-7:10.27
34458:22.16
VrBr004:29.13
VRID9:30.12
VrBr-SSR032:31.83
VrBr-SSR033:32.39
VRID5:33.38
VRID1:33.95
VrBr017:34.09
VrBr-SSR037:34.51
VrBr-SSR039:35.93
VrBr022:39.22
VrBr023:44.62
VrBr024:54.87
VrBr028:61.14
VrBr029:63.34
VrBr030:67.00
VR256:84.12
