# Supplementary material for: Novel Alleles of Two Tightly Linked Genes Encoding Polygalacturonase-Inhibiting Proteins (VrPGIP1 and VrPGIP2) Associated with the Br Locus That Confer Bruchid (Callosobruchus spp.) Resistance to Mungbean (Vigna radiata) Accession V2709
Source: Front Plant Sci. 2017 Sep 28;8:1692. doi: 10.3389/fpls.2017.01692 (PMC5625325; doi:10.3389/fpls.2017.01692)

Supplementary Figure S3 Phylogenic tree depecting relationship of VrPGIP1 and VrPGIP2 from mungbean to other PGIPs from common bean, soybean,chickpea, and Medicago.

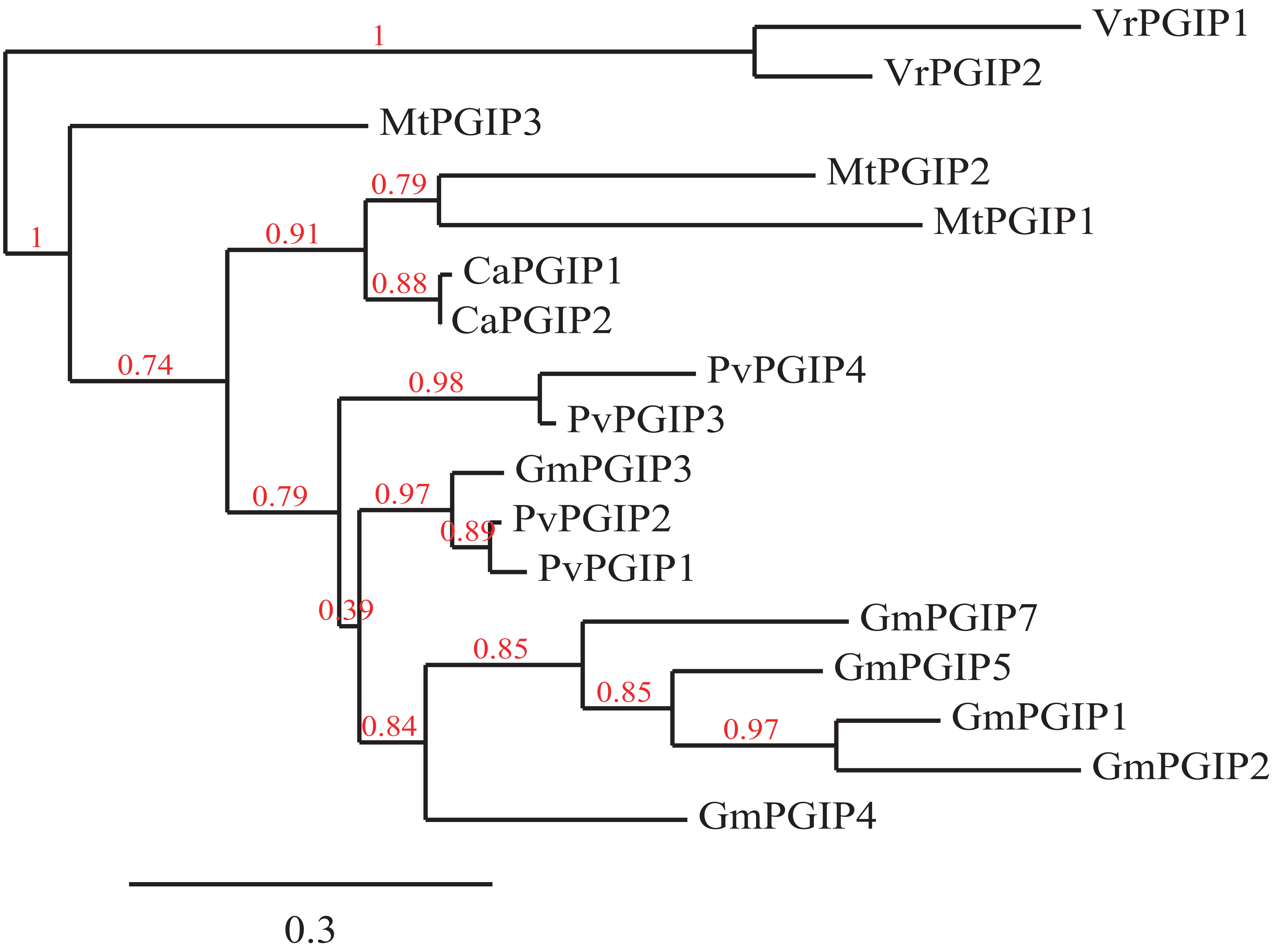

Supplement: Supplementary file 5 [file Image3.PDF]
